# Editorial for Special Issue “Animal Viral Infectious Diseases”

**DOI:** 10.3390/microorganisms14030717

**Published:** 2026-03-23

**Authors:** He Zhang

**Affiliations:** State Key Laboratory of Animal Disease Control and Prevention, Harbin Veterinary Research Institute, Chinese Academy of Agricultural Sciences, Harbin 150001, China; zhanghe01@caas.cn; Fax: +86-451-51997155

Animal viral infectious diseases continue to pose a persistent threat to the development of global animal husbandry and animal health. Since the beginning of the 21st century, significant progress has been made in both the depth of research and the technological approaches within the field of animal virology. Elucidating how animal viral proteins hijack host cell systems to complete invasion, replication, and transmission, as well as how the host immune system recognizes and eliminates viral infections, represents the core scientific question for understanding viral pathogenic mechanisms. Breakthroughs in this fundamental research lay the groundwork for developing clinical solutions. Concurrently, early, rapid, and accurate pathogen detection serves as a critical defense line to block epidemic transmission, and the development of innovative detection tools and methods plays a vital role in controlling outbreaks. All of this content is covered in the Special Issue titled “ Animal Viral Infectious Diseases”. In the study of host–virus interactions, significant progress has been made in elucidating both viral immunosuppression and pathogenic mechanisms. Tengfei Shi et al. (Contribution 1) investigated how viruses weaken immune responses by interfering with the development of host immune cells. Through single-cell mRNA sequencing of mouse splenocytes, they revealed a novel mechanism by which Porcine Circovirus Type 2 (PCV2) impairs vaccine immunogenicity. This occurs by disrupting the interactions between light-zone germinal center B cells and dendritic cells, macrophages, and follicular helper T cells, thereby inhibiting the differentiation of B cells into memory B cells and plasma cells. Zhenkai Dai et al. (Contribution 2) identified the S2 subunit of the spike glycoprotein as the key determinant conferring duodenal tropism to infectious bronchitis virus CSL strains (IBV CSL). Comparative pathogenicity studies in specific-pathogen-free (SPF) chickens and reverse genetics experiments constructed using the non-enterotropic D90 backbone demonstrated that recombinant viruses carrying the CSL-S2 gene, but not those carrying CSL-S1, could efficiently replicate and induce inflammation in the duodenum, exhibiting a phenotype identical to the wild-type CSL strain. In contrast, renal tropism was independent of the S2 subunit. This provides a clear target for the development of next-generation vaccines against enteropathogenic IBV. Laura V. Solarte-Murillo et al. (Contribution 3) compared Orthoreovirus piscis genotype 3 (PRV-3) infection in coho salmon with Orthoreovirus piscis genotype 1 (PRV-1) infection in Atlantic salmon using an erythrocyte infection model. PRV-3 infection of coho salmon erythrocytes induced significant metabolic disruption, apoptosis, and activation of the type I interferon pathway, whereas PRV-1 infection of Atlantic salmon erythrocytes elicited only a mild response. These findings highlight species-specific differences in erythrocyte responses to Orthoreovirus piscis (PRV) infection and provide new insights into the pathogenesis of PRV-3 and PRV-1. Regarding clinical therapeutic strategies, Xiulei Cai et al. (Contribution 4) focused on leveraging knowledge of host–virus interactions to develop novel vaccines. Addressing the challenge of activating mucosal immunity due to the enteric transmission characteristics of porcine epidemic diarrhea virus (PEDV), they found that a recombinant Lactococcus lactis (L. lactis) strain expressing the PEDV S1 and M proteins, administered orally, significantly increased the proportion of CD3+CD4+ and CD3+CD8+ double-positive cells in BALB/c mice and newborn piglets. Concurrently, it notably elevated levels of serum IgG, IgA, and mucosal SIgA, demonstrating promising potential as an oral vaccine vector.

In recent years, the epidemiological situation of infectious viral pathogens in various livestock populations has become increasingly severe due to accelerated viral mutations, frequent cross-border transmission, and the complexity of host immunoregulatory mechanisms. Yongjie Mei et al. (Contribution 5) conducted a large-scale epidemiological investigation of Porcine Reproductive and Respiratory Syndrome Virus (PRRSV) in eastern China, covering 11 sow farms and 53 fattening farms, with a total of 14,934 samples collected. The results showed that NADC30-like, NADC34-like, and HP-PRRSV are the currently predominant circulating lineages. Furthermore, the Nsp2 genes of multiple NADC34-like strains were highly correlated with those of NADC30-like strains, indicating that they are essentially recombinant viruses. This study also provided the first assessment of the pathogenicity of a representative NADC34-like virus isolated in China. All infected sows experienced abortion, with 100% of the aborted piglets being stillborn and exhibiting high viral loads, confirming the high pathogenicity of this strain for sows. Certain animal viruses capable of infecting humans not only pose a serious threat to animal health but also lead to significant public health problems. Since the emergence of the Junín virus in 1953, pathogenic New World arenaviruses have been a persistent issue, causing acute viral hemorrhagic fevers and neurological complications that result in severe morbidity and mortality. Alexander V. Alvarado et al. (Contribution 6) provided a comprehensive review of publicly reported animal models for infection with pathogenic New World arenaviruses, discussing the advantages and disadvantages of each model. Given the lack of licensed treatments and vaccines, animal infection models that accurately replicate the symptoms of human disease are critically important for the development of medical countermeasures.

Concurrently, the timely detection of animal infectious viruses is particularly critical for preventing disease outbreaks, with novel detection methods enabling faster and more accurate virus diagnosis. In terms of innovation in detection technology, Jorge Morales et al. (Contribution 7) developed a set of improved primers and probes incorporating degenerate sites to address the challenge posed by new variants of African horse sickness virus (AHSV). Validated collaboratively by WOAH reference laboratories, the improved method effectively detects nucleic acid from a wide range of AHSV strains and clinical samples, including the new variants, providing a reliable tool for the surveillance and control of African horse sickness. Fuxing Hao et al. (Contribution 8), targeting the complex and diverse pathogenic spectrum of bovine respiratory disease complex (BRDC)—which is often characterized by mixed infections—developed a novel multiplex real-time PCR assay. This assay can simultaneously detect eight major pathogens: BVDV, BPIV3, BRSV, BCoV, M. bovis, P. multocida, M. haemolytica, and IBRV. The method exhibits no cross-reactivity among the targets, achieves a detection limit as low as five copies per reaction, and has a coefficient of variation below 2%. Testing results from 1012 clinical samples on two farms in Jiangsu Province revealed a high proportion of mixed infections, highlighting the clinical value of this multiplex detection method. This Special Issue brings together research advances in the field of animal virology concerning host–virus interactions, viral epidemiology and pathogenicity assessment, and the development of novel detection technologies. It also points the way for future work, directions that are likely to open new opportunities for the formulation of prevention and control strategies and for clinical application research concerning animal viral infectious diseases.

